# Exergy Analysis and Human Body Thermal Comfort Conditions: Evaluation of Different Body Compositions

**DOI:** 10.3390/e20040265

**Published:** 2018-04-10

**Authors:** Matheus Martinez Garcia, Rafael Yoshimori Une, Silvio de Oliveira Junior, Carlos Eduardo Keutenedjian Mady

**Affiliations:** 1 School of Mechanical Engineering, University of Campinas, Mendeleyev St., 200, 13083-970 Campinas-SP, Brazil; 2 Polytechnic School of the University of São Paulo, Av. Luciano Gualberto 380, 05508-010 São Paulo, Brazil

**Keywords:** exergy analysis, human thermal model, thermal comfort conditions

## Abstract

This article focuses on studying the effects of muscle and fat percentages on the exergy behavior of the human body under several environmental conditions. The main objective is to relate the thermal comfort indicators with exergy rates, resulting in a Second Law perspective to evaluate thermal environment. A phenomenological model is proposed of the human body with four layers: core, muscle, fat and skin. The choice of a simplified model is justified by the facility to variate the amount of mass in each tissue without knowing how it spreads around the body. After validated, the model was subjected to a set of environmental conditions and body compositions. The results obtained indicate that the area normalization (Watts per square meter) may be used as a safe generalization for the exergy transfer to environment. Moreover, the destroyed exergy itself is sufficient to evaluate the thermal sensation when the model is submitted to environmental temperatures lower than that considered for the thermal neutrality condition (and, in this text, the thermal comfort) . Nevertheless, for environments with temperatures higher than the calculated for the thermal neutrality, the combination of destroyed exergy and the rate of exergy transferred to the environment should be used to properly evaluate thermal comfort.

## 1. Introduction

The Laws of Thermodynamics describe different types of phenomena in several research areas. Common examples are found in chemistry, astrophysics, materials science and engineering. The Second Law of Thermodynamics demonstrates that there must be an impact in the environment wherever a non-equilibrium is present. As discussed by [1], it is this unbalance that guarantees life. A few decades later [2] demonstrated that all living beings tend to a minimum entropy production level.

Several Thermodynamic approaches to biological systems have been performed in the past decades. To cite some, there is the study of a single cancerous cell [3], protocols in hypothermia techniques [4], and an analysis of physical activities [5,6,7]. There is also a review article gathering these new applications as in Ozilgen [8].

In mechanical engineering, one possible application of Thermodynamics is the evaluation of thermal comfort conditions. Rupp et al. [9] performed a review of thermal comfort in build environment and pointed out that the exergy analysis may bring new information to this research field.

There are three main approaches regarding the relation of the destroyed exergy and thermal comfort conditions. Some authors [10,11,12,13,14] obtained that only a certain combination of environmental conditions results in minimal destroyed exergy, and these conditions coincide with points of thermal comfort. Other authors [15,16,17,18,19,20,21] evaluated the effect of the internal and external environmental conditions in the destroyed exergy of the body and in the exergy consumption in buildings (relating these results with the body exergy consumption). Eventually, [22,23,24,25] assessed the destroyed exergy and the exergy efficiency of the body. Their results indicates that the destroyed exergy may be minimal at points that do not indicate thermal comfort. Nevertheless, at these points the exergy transfer to the environment is minimal and, as consequence, a natural reasoning is to use these two physical quantities in order to assess thermal comfort conditions. It is important to state that due to the difference in the methods the results of destroyed exergy for different references differ in one order of magnitude. This difference may be explained by the exergy input of the model, which is considered as a reaction rate (in energy and exergy basis) [23]; or only the effect of heat transfer, depending on the type of irreversibility’s chosen to be evaluated. Therefore, when the metabolism is considered only as a heat transfer rate, the exergy associated with this physical quantity results in lower values of destroyed exergy.

It is common to divide the exergy rates by the skin area in order to generalize the relation between the exergy rates and the thermal comfort. The human body, however, does not necessarily have a The human body, however, does not necessarily have a linear response with its area (although this is the best normalization for physiological parameters related to heat transfer) as its tissues have very singular properties, like the high muscle metabolism and the thermal insulation characteristic (and low metabolism) of fat. Hence, the effects of the tissues should be considered for a proper analysis of the human body, as they change the body response even if the surface area is the same. The objective of this work is to analyze the effects of the body composition in the destroyed exergy and in the exergy transfer to environment. This article is a logical continuation of the group previous articles [23,24,25], where it was obtained that in several cases the minimal destroyed exergy occurs at the same point of thermal comfort conditions. Nevertheless, there were points (high temperature and low relative humidities) in which the detroyed exergy was minimal, although the thermal comfort condition was not expected. The exergy transfer to environment ($B_{env}$) was high at these points, indicating that this physical quantity could play an important role in the search for thermal comfort conditions. This article intends to make a comparison between these two quantities, the internal ($B_{d}$) and the external ($B_{env}$—destroyed in the environment) irreversibilities, and traditional thermal comfort indexes.

## 2. Methods

### 2.1. Thermodynamic model

**Figure 1:**
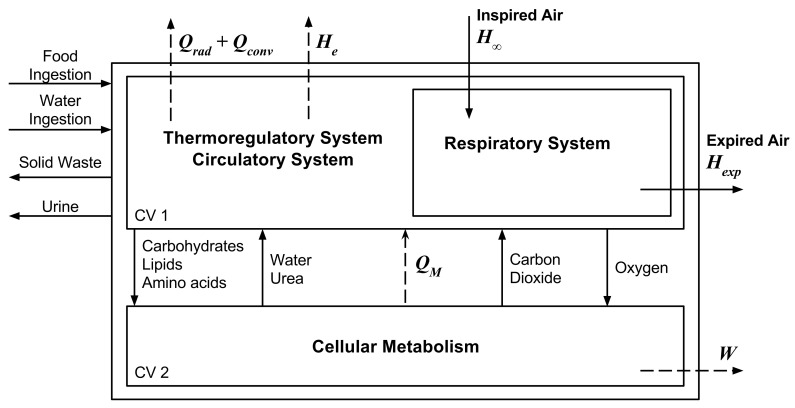
Thermodynamic human body model with heat and enthalpy transfer rates to environment. Based (and adapted) from [26].

The human body thermal model developed for this article is based on the model used by [24], described in [26]. The human body is modelled as two control volumes, as shown in Figure 1. CV1 represents the macroscopic part of the body, while CV2 represents the cellular metabolism. Focusing on CV2, the oxidation of nutrients during the cellular respiration generates two therms: the heat, ${\dot{Q}}_{M}$, transferred to the body (CV1) to keep its temperature in a narrow range, and the work, $\dot{W}$, used in activities. CV1 interacts with the environment, thus it is considered the convection, ${\dot{Q}}_{conv}$, and the radiation, ${\dot{Q}}_{rad}$, heat transfers from the skin. There is also an enthalpy flow rate to environment, ${\dot{H}}_{e}$, due the vaporization of water at the skin surface. The respiratory system operation generates an enthalpy flow rate entering the body, ${\dot{H}}_{0}$, and an enthalpy flow rate leaving the body, ${\dot{H}}_{exp}$.

The energy balance of a control volume involving CV1 and CV2 results in Equation (1). In this equation the metabolism, $\dot{M}$, is considered an enthalpy variation over time of the reactions of oxidation inside the control volume. The enthalpy flow rate caused by food and water ingestion and by wastes and urine are disregarded, since it is considered that the body does not have any of these mass transfers during the period of study. The exergy balance of the same control volume is shown in Equation (2). The terms inside the parentheses in Equation (1) are usually agglutinated in one term called energy transfer to environment, ${\dot{E}}_{env}$, and in Equation (2) , in one term named exergy transfer to environment, ${\dot{B}}_{env}$.

(1) $$\frac{dU}{dt}=\dot{M}-\dot{W}-\left({\dot{Q}}_{conv}+{\dot{Q}}_{rad}+{\dot{H}}_{e}+\Delta {\dot{H}}_{resp}\right)$$

(2) $$\frac{dB}{dt}={\dot{B}}_{M}-\dot{W}-\left({\dot{B}}_{conv}+{\dot{B}}_{rad}+{\dot{B}}_{e}+\Delta {\dot{B}}_{resp}\right)-{\dot{B}}_{d}$$

### 2.2. Energy and Exergy Metabolisms

Mady and Oliveira Junior [26] evaluated the enthalpy and exergy changes of the nutrients’ reaction of oxidation during the cellular respiration, based in the article of [27]. The nutrients chosen by the authors were glucose, as a representative of carbohydrates, palmitic acid, representing the lipids and an amino-acid with average composition ($C_{4.98}H_{9.8}N_{1.4}O_{2.5}$) representing the proteins. It was considered that the glucose and the palmitic acid suffer complete oxidations in the body, while the amino-acid oxidizes only until the formation of urea.

The energy metabolism can be calculated using Equation (3), while the exergy metabolism is obtained by applying Equation (4). In this article the energy metabolism is given by the body composition, and its calculation will be explored forward (Section 2.5). Equation (3), is used herein to calculate the nutrients consumption rate, necessary to obtain the exergy metabolism and the respiratory enthalpy exchange.

(3) $$\dot{M}=-\left({\dot{m}}_{carb}\Delta h_{carb}+{\dot{m}}_{prot}\Delta h_{prot}+{\dot{m}}_{lip}\Delta h_{lip}\right)$$

(4) $${\dot{B}}_{M}=-\left({\dot{m}}_{carb}\Delta b_{carb}+{\dot{m}}_{prot}\Delta b_{prot}+{\dot{m}}_{lip}\Delta b_{lip}\right)$$

Applying the hypothesis made by [28], that there is a daily excretion of 12 g of nitrogen in urea produced by amino-acids oxidation, and using the equations of the reactions of oxidation, it is possible to obtain the mass of amino-acids consumed in one day and its rate, considered constant. To obtain the carbohydrate and lipid consumption rates it is necessary to solve the remaining system, composed by Equation (3), the respiratory coefficient, RQ, and the oxidation reaction equations. The RQ represents the ratio between the carbon dioxide generation and the oxygen consumption during the respiration at volumetric basis (or molar basis using the ideal gas model). As discussed in [29] a typical value of RQ is 0.83 for a person in daily activities.

### 2.3. Energy Transfer to Environment

The body interacts with the environment through the convection heat transfer, the radiation heat transfer, the sweat vaporization (and water diffusion) enthalpy transfer and the respiratory enthalpy variation. The equations used to obtain these terms were well explored in literature and can be found in [23,24,29,30]. The body is considered naked and in an hypothetical environment, where the mean radiant temperature ($T_{mr}$) is the same as the air temperature ($T_{a}$), therefore, being the same for the operative temperature ($T_{o}$).

### 2.4. Exergy Transfer to Environment

The environmental conditions are adopted as reference during the calculation of the exergy transfer to environment. Therefore, for a given environment, the reference of the relative humidity ($\phi_{0}$), atmospheric pressure ($P_{0}$) and operative temperature ($T_{0}=T_{o}$) are determined. The exergy transfer rates associated with the convection and radiation heat transfers are evaluated as Equations (5) and (6), respectively. The evaporative exergy flow rate to environment is given by Equation (7). The exergy of the expired air is calculated using Equation (8). Note that ${\dot{B}}_{0}=0$ due the use of the environmental conditions as reference ($T_{0}$).

(5) $${\dot{B}}_{conv}=\left(1-\frac{T_{0}}{T_{s}}\right){\dot{Q}}_{conv}$$

(6) $${\dot{B}}_{rad}=\left(1-\frac{T_{0}}{T_{s}}\right){\dot{Q}}_{rad}$$

(7) $${\dot{B}}_{e}={\dot{m}}_{e}\left[h_{fg}-T_{0}s_{fg}+T_{0}R_{g}ln\left(\frac{p_{g,skin}}{p_{g,0}}\right)\right]$$

(8) $${\dot{B}}_{exp}=\sum_{i=1}^{4}{\dot{m}}_{i}\left[c_{p,i}\left(T_{i}-T_{0}-T_{0}ln\left(\frac{T_{i}}{T_{0}}\right)\right)+T_{0}R_{i}ln\left(\frac{p_{exp,i}}{p_{0,i}}\right)\right]$$

where, in these equations $T_{0}$ is the reference environmental temperature, $T_{s}$ is the skin temperature, $h_{fg}$ is the enthalpy of vaporization of water at the skin temperature, $s_{fg}$ is the entropy of vaporization of water at the skin temperature, $R_{g}$ is the gas constant of the water, ${\dot{m}}_{e}$ is the rate of sweat eliminated through skin, $p_{g,skin}$ of the partial pressure of water vapor in the skin, $p_{g,0}$ is the partial pressure of water vapor in the environment; these equations are analysed in [23,24]. For Equation (8) the index *i* refers to the gases of the respiration, which is oxygen, carbon dioxide, water vapor and nitrogen. In this equation $p_{exp,i}$ is the partial pressure of the expired gas *i* and $p_{0,i}$ is the partial pressure of the gas *i* in the envinroment.

### 2.5. Human Thermal Model

**Figure 2:**
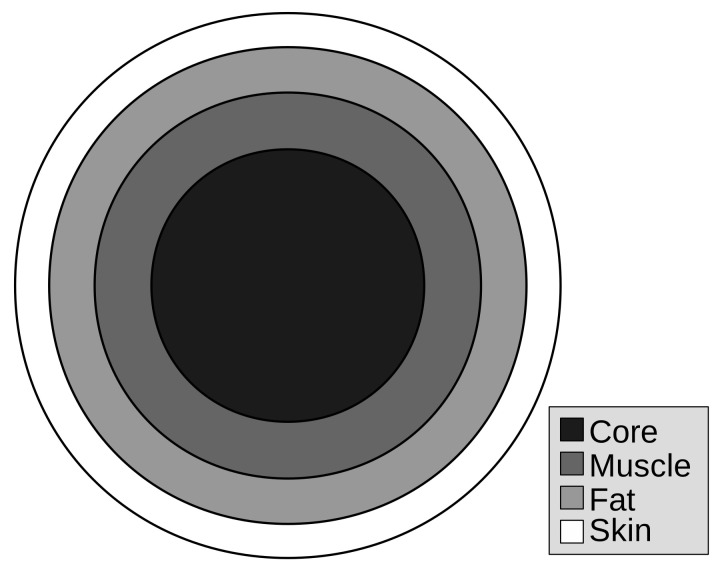
Phenomenological representation of the human body with four layers representing the tissues (core, muscle, fat and skin) This simplification makes it possible to variate some tissues volume in order to evaluate its effects on the energy and exergy behaviour of the human body and, therefore, in thermal comfort conditions, based on [30,31].

To obtain the temperature profile of the body, essential for the application of the exergy analysis, it is necessary to develop a human body heat transfer model. The model that follows in this section is a simplification of [30]. The human body in this article is considered as a four-layer cylinder, which layers consist, from inner to outer, in core, muscle, fat and skin. Figure 2 indicates a representation of the human body as a cylinder where each layer is represented, accordingly.

The initial geometry of the body was calculated using the data for the standard man and was based on the previous work of [31]. Werner and Buse [32] present some physical and thermal properties of many organs and tissues of the standard man, and define him as a man with 1.76 m height, 67 kg weight, 1.8 m${}^{2}$ area and 67 dm${}^{3}$ volume. Since the body is modelled as a cylinder, it is impossible to keep all the parameters of the standard man. Tests made with the model showed that the temperature profile is closer to reality if the height of the standard man is maintained instead of its area. Therefore, the height of the model is fixed at L=1.76 m, and its area and volume should be calculated for each geometry using geometrical relations.

Most of the human body blood is distributed along tissues and organs in the data provided by [32]. Considering that the volume of this element is the same than provided by the authors, their mass must be corrected. The correction is made by estimating the total blood volume using the equation proposed by Nadler [33], and considering that this blood is equally distributed along all organs and tissues. The volume of the layers is calculated by adding the volume of each element that composes it. Note that, after the correction, the blood volume is disregarded for the body volume calculation, but its mass should be taken into account. Table 1 demonstrates these weightings and the results of each tissue thermophysical properties.

It is considered that there is not a variation of the mass of the organs (or volume) as a function of the increase/decrease of lean/fat body masses. Therefore, the core layer has a constant radius, $r_{c}=6.078$ cm. Besides that, the skin layer is considered always with the same thickness, 0.28 cm, independent of the body composition. These values were calculated using the data for the standard man. The muscle and fat layers are free to vary. The body metabolism is then obtained by summing the metabolism of each layer, as shown in Equation (9). The blood volume, $V_{b}$ (in m${}^{3}$), should be evaluated for every body composition using Equation (10), obtained from [33].

(9) $${\dot{M}}_{bas}=V_{c}{\dot{M}}_{bas,c}^{{}^{\prime \prime \prime }}+V_{m}{\dot{M}}_{bas,m}^{{}^{\prime \prime \prime }}+V_{f}{\dot{M}}_{bas,f}^{{}^{\prime \prime \prime }}+V_{s}{\dot{M}}_{bas,s}^{{}^{\prime \prime \prime }}$$

(10) $$V_{b}=\left(0.3669L^{3}+0.03219bm+0.6041\right)10^{-3}$$

The human body mechanisms in order to adapt to the environment where it is submitted is called thermoregulation or control system of the body. These mechanisms exist in order to control the heat exchanges with the environment, preserving the internal temperatures as close as possible of the normothermia conditions [4]. The human body control system is activated when the body departs from the thermal neutrality condition (for the model, thermal neutrality can be interpreted as the same of thermal comfort conditions). The thermal neutrality condition is obtained by submitting the model set for the standard man (naked) to an environment at 30 ${}^{\circ }$C and 50% relative humidity, as indicated by [30]. The model developed in this article considers that the temperature profile generated by this condition represents the thermal neutrality for every composition studied. In other words, it means that the temperature profile of the model for the thermal neutrality condition does not change from person to person. This profile consists in $T_{c}^{0}=37.6$ ${}^{\circ }$C, $T_{m}^{0}=37.0$ ${}^{\circ }$C, $T_{f}^{0}=35.9$ ${}^{\circ }$C, $T_{s}^{0}=35.4$ ${}^{\circ }$C and $T_{b}^{0}=37.4$ ${}^{\circ }$C.

The thermoregulatory system is composed by a sweat modeling, adapted from [35], a vasoconstriction and vasodilatation model, adapted from [36], and a shivering model, obtained from [37].

The energy balance of the core layer is given by Equation (11). Note that the enthalpy variation due the respiration is considered uniformly distributed all along the core volume. In this equation, $\rho_{c}$ is the specific mass of the core, $V_{c}$ is the volume of the core, $q_{c\to m}$ is the heat transfer between core and muscle and $q_{b\to c}$ is the heat transfer between the blood and tissue in the small vessels according to the model proposed by [38]

(11) $$\rho_{c}V_{c}c_{p,c}\frac{dT_{c}}{dt}=-q_{c\to m}+q_{b\to c}+{\dot{M}}_{c}^{{}^{\prime \prime \prime }}V_{c}-\Delta {\dot{H}}_{resp}$$

The energy balances of the muscle and fat layers have the same form, and are given by Equations (12) and (13), respectively.

(12) $$\rho_{m}V_{m}c_{p,m}\frac{dT_{m}}{dt}=q_{c\to m}-q_{m\to f}+q_{b\to m}+{\dot{M}}_{m}^{{}^{\prime \prime \prime }}V_{m}$$

(13) $$\rho_{f}V_{f}c_{p,f}\frac{dT_{f}}{dt}=q_{m\to f}-q_{f\to s}+q_{b\to f}+{\dot{M}}_{f}^{{}^{\prime \prime \prime }}V_{f}$$

The skin layer energy balance is given by Equation (14).

(14) $$\rho_{s}V_{s}c_{p,s}\frac{dT_{s}}{dt}=q_{f\to s}+q_{b\to s}+{\dot{M}}_{s}^{{}^{\prime \prime \prime }}V_{s}-{\dot{Q}}_{conv}-{\dot{Q}}_{rad}-{\dot{H}}_{e}$$

Eventually the First Law of Thermodynamics is also applied to a central reservoir of blood [30]. The heat exchange between blood and tissue ($q_{b\to i}$) is given by Pennes’ model [38]. The heat exchange between tissues, $q_{i\to j}$, is calculated using the thermal conductivities weighted by the volume of the layers in contact, and the temperatures of the layers are considered constant. To solve the differential equations a C++ program was developed for the explicit Euler method.

The variation of the environmental conditions causes the variation of the body internal temperature profile over time [23], but herein the focus is given only in the steady-state points, obtained when the body attains an equilibrium state with the new environment. In other words, for the body perspective, steady state may be defined when there is no variation of the temperature of each tissue over time. The transient conditions may be used in future analysis where modifications in the actual environment may be evaluated. It is important to highlight that in the period considered, minutes of simulations, the water mass lost in sweat is negligible (larger periods of time, such as one day this is not true), nevertheless its energy must be considered.

### 2.6. Thermal Comfort Indexes

Although it is difficult to obtain a rational definition of thermal comfort conditions [29], for the model perspective it is possible to define the thermal comfort as the same of thermal neutrality (since the environment analysed will not have any local discomfort).

Fanger [39] proposed the Predicted Mean Vote (PMV) and the Predicted Percent Dissatisfied (PPD) as alternatives to evaluate the environment aiming to predict a “satisfactions” or “dissatisfaction” with the thermal environment. The PMV index uses the Ashrae scale [29], a scale that is defined on integers from −3 to 3. The negative values of PMV represent the so called “cold" sensation while the positive values represent the “hot” sensation. The further PMV is from zero, the bigger the thermal discomfort.

In this article, the predicted mean vote (PMV) was calculated according to Fanger method as indicated in Equation (15). Where, according to [29] “L is the thermal load on the body, defined as the difference between internal heat production and heat loss to the actual environment for a person hypothetically kept at comfort values”. Therefore, the predicted mean vote evaluate the influence of control system in the energy balance.

(15) $$PMV=(0.303exp\left(-0.036\dot{M}\right)+0.028)L$$

In order to quantify the number of people who are not satisfied with the environment Equation (16) indicates the predicted percent dissatisfied (PPD) [29].

(16) $$PPD=100-95exp(-\left(0.03353PMV^{4}+0.2179PMV^{2}\right))$$

These physical quantities will be compared with the exergy analysis results (destroyed exergy and exergy transfer to the environment).

## 3. Results and Discussion

The results obtained during the simulations will be divided in three subsections. The first subsection introduces the model validation, where the results are compared with the literature. The second subsection presents the exergy destruction and the exergy transfer to environment for a set of body constitutions. The last subsection focus on relating the thermodynamic data with thermal comfort conditions.

### 3.1. Validation of the Exergy Behavior of the Human Thermal Model

**Figure 3:**
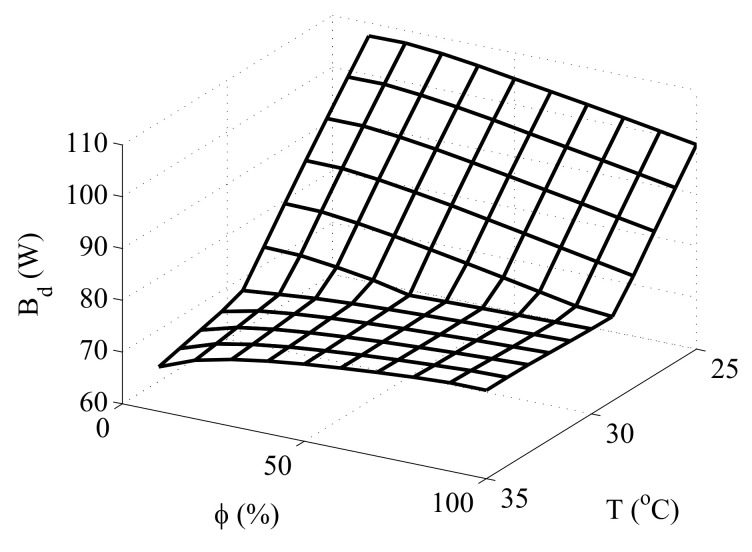
Exergy destruction rate of the human body as a function of environmental parameters obtained in [40] and used as basis to validate the exergy behaviour of the simplified model.

Mady et al. [23], working with the model of 15 cylinders developed by [30], obtained a Figure for the exergy destruction rate as a function of operative temperature and relative humidity. Figure 3 was obtained using the model developed in this article for the same anatomy. Analysing Figure 3, it is possible to note that both models have a similar trend, although the Figure proposed in [23] presents some non-linearity in the model response. Besides that, both models resulted in similar values of destroyed exergy. It can be noted in both a decrease of the destroyed exergy at environments with temperatures higher than 30 ${}^{\circ }$C and humidities lower than 50%, and an increase in this physical quantity at environments with temperatures lower than 30 ${}^{\circ }$C, specially for relative humidities lower than 50%. The only significant difference among the curves takes place for high temperatures and relative humidities, where the Figure proposed in [23] takes into account the Q${}_{10}$ effect, whereas in Figure 3, for the sake of simplicity, this effect was not considered.

### 3.2. Exergy Analysis and Body Composition

**Figure 4:**
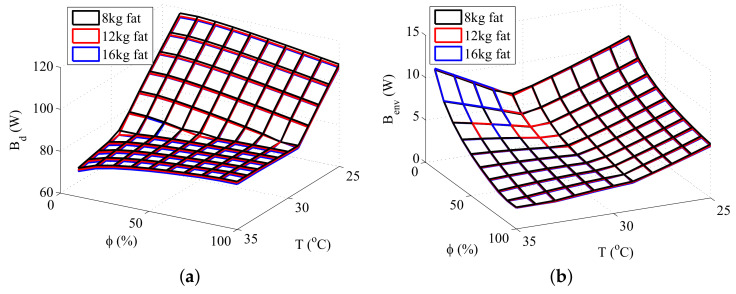
Exergy rates for a body mass of 80 kg: (**a**) Destroyed Exergy ($B_{d}$); (**b**) Exergy transfer to environment ($B_{env}$).

**Figure 5:**
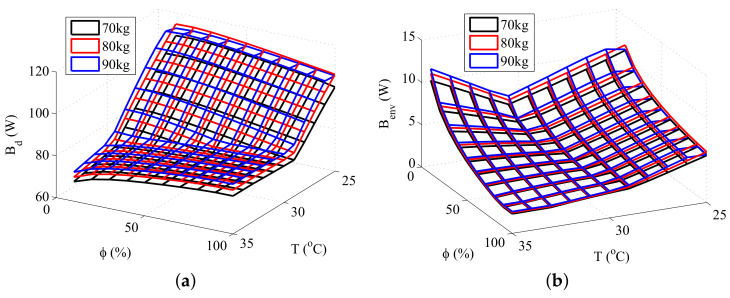
Exergy rates for 30 kg of muscle and different fat masses: (**a**) Destroyed Exergy ($B_{d}$); (**b**) Exergy transfer to environment ($B_{env}$).

**Figure 6:**
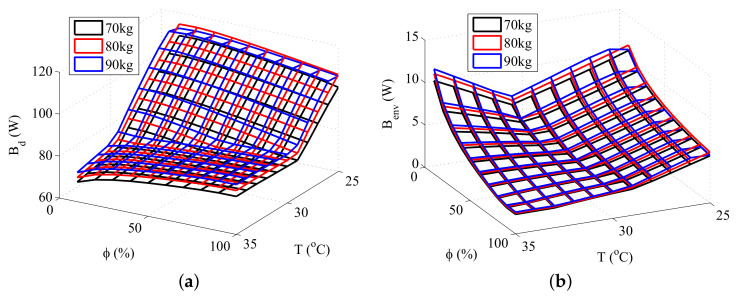
Exergy rates for a fat mass of 15 kg and different muscle masses: (**a**) Destroyed Exergy ($B_{d}$); (**b**) Exergy transfer to environment $B_{env}$.

Three types of simulations were performed in order to study the interaction between exergy quantities and body composition. The first simulation consisted of maintaining the total body mass constant while varying the amount of fat (therefore, causing a variation in muscle and fat layers), the second in modifying the amount of fat while keeping the muscle mass constant, and the third in modifying the quantity of muscle keeping the fat mass constant. The results obtained are shown in Figure 4, Figure 5 and Figure 6, respectively.

Figure 4 shows that there is not a significant difference in the exergy behavior of the body if the total mass is kept constant. This may not be true for higher (or lower) body masses, where the muscle and fat effects may not be under balance.

Figure 5 and Figure 6 shows that both muscle and fat contributes to the destruction of exergy and to the transfer of exergy to environment, although an increase in the muscle mass results in a higher increase in these thermodynamic properties than the same increase in the fat mass. This result is related to the rise of the body metabolism, and occurs because the muscle layer requires more energy than the fat layer. The rise of the metabolism results in an increase of the oxidation rates, and, as consequence, in higher irreversibilities and exergy transfers to environment. Moreover, at low temperatures there is also the muscle shivering, which requires an even higher oxidation rate, hence being responsible for the main increase in the destroyed exergy of the body at these environments.

These figures also demonstrates that the minimum exergy destruction occurs for environments with high temperatures and low relative humidities, independent of the body composition. The combination of high temperatures and low humidities is found in deserts, where the body is far from the thermal comfort conditions. Therefore, another exergy-based index must be used along with the destroyed exergy to properly evaluate the environment. Mady, C.E.K, et al. [24] proposes that points of simultaneous minimal $B_{d}$ and $B_{env}$ may be related to thermal neutrality, and, as consequence, to thermal comfort conditions.

### 3.3. Exergy Analysis and Thermal Comfort

**Figure 7:**
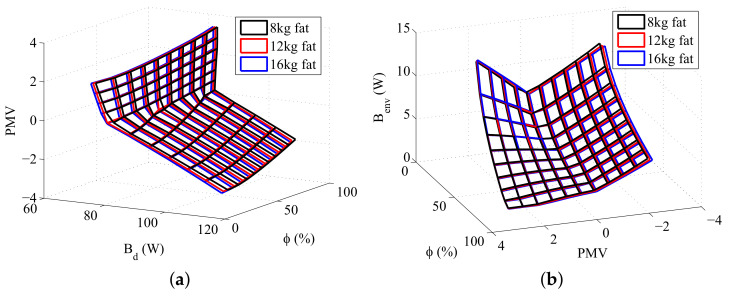
The relation between some exergy rates and PMV for a body mass of 80 kg: (**a**) Destroyed Exergy ($B_{d}$); (**b**) Exergy transfer to environment ($B_{env}$).

**Figure 8:**
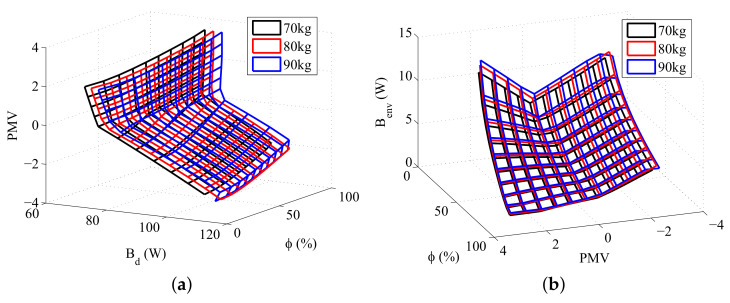
The relation between some exergy rates and PMV for a muscle mass of 30 kg: (**a**) Destroyed Exergy ($B_{d}$); (**b**) Exergy transfer to environment ($B_{env}$).

**Figure 9:**
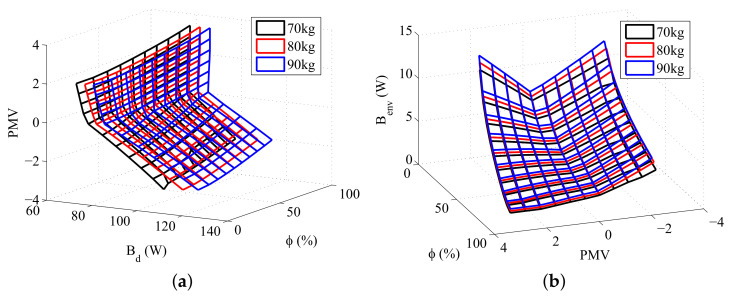
The relation between some exergy rates and PMV for a fat mass of 15 kg: (**a**) Destroyed Exergy ($B_{d}$); (**b**) Exergy transfer to environment ($B_{env}$).

The data required to calculate the PMV according to Fanger Method is obtained during the resolution of the energy and exergy balances, making it possible to evaluate this index for the set of environments selected. Furthermore, it is possible to compare the exergy destruction, or the exergy transfer to environment, with the PMV for different situations. Figure 7, Figure 8 and Figure 9 illustrates these comparisons.

Analysing Figure 8b and Figure 9b it is notable that, for the same PMV, the exergy transfer to environment is higher if the body has more mass, indicating that the exergy transfer to environment increases with the body metabolism. Figure 6b confirms this relation since, for the most part of the graph, the body with more muscle, and consequently, higher metabolism, has the biggest exergy transfer to environment. The metabolism is an energy variation inside the body that must be dissipated in order to keep the body internal temperatures at the same levels. It is natural then that a body with more reactions rate has a higher energy transfer to environment, and as consequence, higher exergy transfer to environment. The Figure 6a, Figure 7a and Figure 8a indicates that the exergy destruction also increases with the metabolism for the same PMV. The metabolic energy comes from oxidation reactions that present irreversibilities, as result, when the body mass (and its metabolism) rises, the exergy destruction increases, since there are more irreversible processes inside the body. Note that in Figure 7b there is an inversion of the graph for high PMVs and low humidities, and the body with the lowest metabolism (most fat) has the highest $B_{env}$, which is an unexpected result.

**Figure 10:**
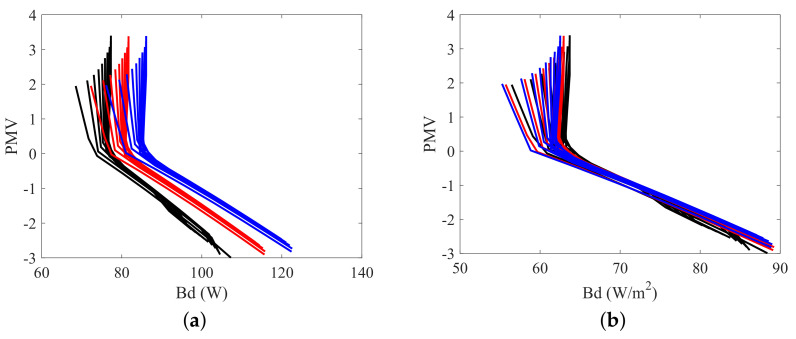
The relation between PMV, some exergy indexes and relative humidity for body masses of 70, 80 and 90 kg, with a constant fat mass of 15 kg: (**a**) PMV as a function of the destroyed Exergy ($B_{d}$); (**b**) PMV as a function of exergy transfer to environment ($B_{env}$).

**Figure 11:**
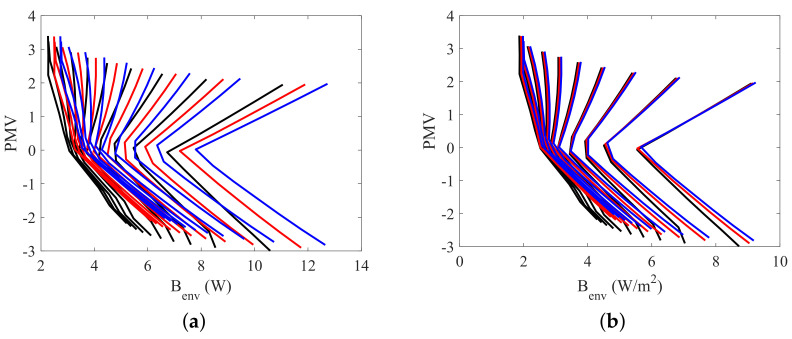
The relation between PMV, some exergy indexes and relative humidity for body masses of 70, 80 and 90 kg, with a constant fat mass of 15 kg: (**a**) PMV as a function of the normalized destroyed Exergy ($B_{d}/A_{s}$); (**b**) PMV as a function of the normalized exergy transfer to environment ($B_{env}/A_{s}$).

**Figure 12:**
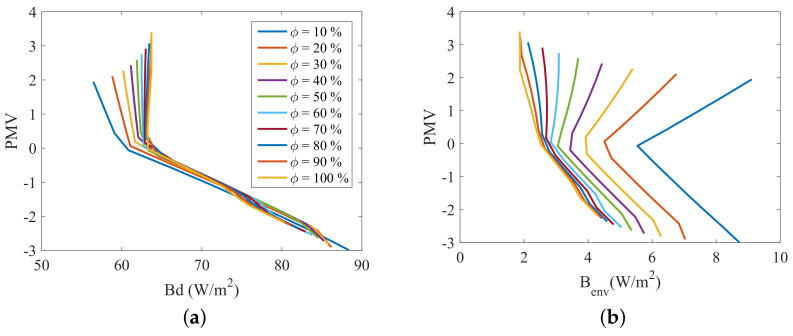
PMV as function of exergy indexes and relative humidity (10<ϕ<100%) for a body mass of 70 kg with a fat mass of 15 kg: (**a**) PMV as function of destroyed Exergy ($B_{d}$); (**b**) PMV as function of exergy transfer to environment ($B_{env}$).

**Figure 13:**
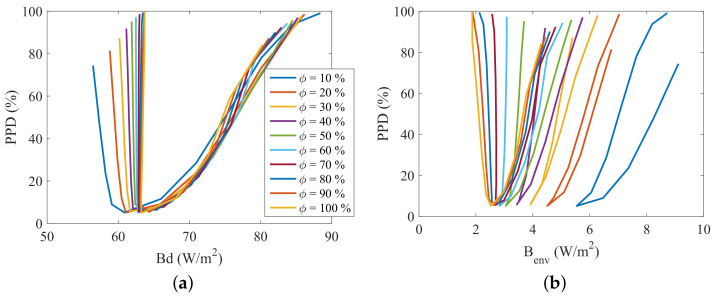
PPD as function of exergy indexes and relative humidity (10<ϕ<100%) for a body mass of 70 kg with fat mass of 15 kg: (**a**) PMV as function of destroyed Exergy ($B_{d}$); (**b**) PMV as function of exergy transfer to environment ($B_{env}$).

The two dimensional plots of Figure 10, Figure 11, Figure 12 and Figure 13 present the relation between the destroyed exergy ($B_{d}$) (or the exergy transfer to environment ($B_{env}$)) and the PMV (or the PPD), for a fixed amount of fat (15 kg). The exergy rates and flow rates are plotted in *W* and in W/m${}^{2}$ in Figure 10 and Figure 11, respectively. The aim is to analyse if the area normalization is a good generalization of the human body response when submitted to different environments. Nevertheless, the composition was fixed and the effect of muscle or fat was evaluated.

Figure 10a indicates that the exergy destruction rate is almost independent of the humidity when the thermal sensation index is negative, and can be approximated to a straight line for a general analysis (there is no diversification of the destroyed exergy trend as a function of relative humidity). As consequence, in this region the use of the destroyed exergy is sufficient to relate with PMV. For these set of conditions the minimum exergy destruction corresponds to the thermal neutrality (and thermal comfort) conditions. However, when PMV is positive it is necessary the use of both: destroyed exergy and exergy transfer to environment to properly judge the environment. For a humidity of 10%, for instance, $B_{d}$ is minimum at PMV>2, but $B_{env}$ has its maximum value for this PMV, while, for a humidity of 100%, $B_{env}$ is minimum for PMV>2, but $B_{d}$ is not minimal at this points.

As a possible rule, for PMV>0, when ϕ≥70% the minimal points of $B_{d}$ corresponds to the thermal comfort conditions, but the minimal of $B_{env}$ does not, and when ϕ<70%, the minimal of $B_{env}$ corresponds to the thermal comfort conditions, but the minimal of $B_{d}$ does not. For PMV<0 the minimal of any of these indicators correspond to the thermal comfort conditions. The results obtained confirm the idea of [24] that the ordinate pair ($B_{d}$, $B_{env}$) must be evaluated in order to properly judge the environment.

Figure 11 shows that the normalized (using the surface area) response of the model is very similar when only one type of tissue (fat or muscle) are modified.

Figure 12 and Figure 13 illustrate the necessity of using both the destroyed exergy rate ($B_{d}$) and the exergy transfer to environment rate ($B_{env}$) to properly evaluate the thermal comfort. It can be seen in this figures that PMV≈0 and PPD≈5% only when both physical quantities are at, or around, the minimum point. This condition is specially important for low humidities. Taking ϕ=10% for instance, when the destroyed exergy is minimal the exergy transfer rate to environment is high, and the person is not at thermal comfort conditions (PMV≈2 for minimal $B_{d}$ at ϕ=10%).

**Figure 14:**
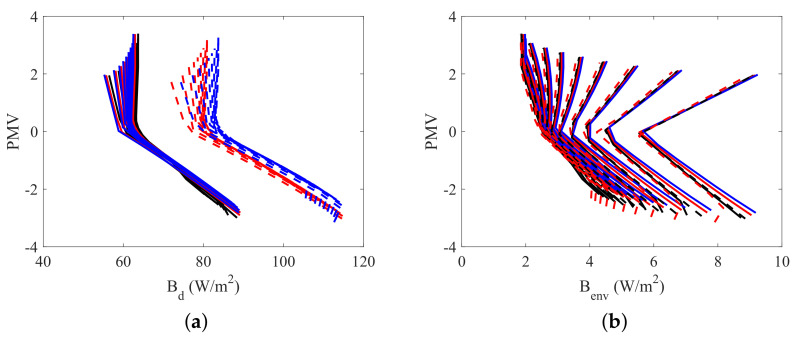
PPD as function of exergy indexes and relative humidity (10<ϕ<100%) for a body mass of 70, 80 and 90 kg with fat mass of 15 kg (continuous line) and for a mass of 80 and 90 kg and muscle mass of 30 kg (dashed lines): (**a**) PMV as function of destroyed Exergy ($B_{d}$); (**b**) PMV as function of exergy transfer to environment ($B_{env}$).

One discussion that must be taken into account is the effect of modifying the muscle or fat mass in the exergy behavior of the body when it is divided by the area W/m${}^{2}$. It is possible to conclude that indeed the exergy rates are not significantly affected by a modification of the tissue independently of the body composition, nevertheless, the destroyed exergy is, since each tissue has its own metabolism. The exergy indexes are plotted in W/m${}^{2}$ in Figure 14, in order to analyse if the area normalization is a good generalization of the human body response when submitted to different environments. Figure 13a shows that modifying the body constitution (specially the muscle mass) affects the destroyed exergy, because of the rise of metabolism, previously explained. The main effect observed is the translation of the curve, which shape is preserved. Moreover, knowing the exergy destruction at the thermal neutrality condition (PMV=0), it is possible to overlap the curves, and obtain a general comportment. Figure 14b shows that the body composition has almost no effect in the exergy transfer to environment, when the indexes are normalized by the skin area. Therefore, both figures represent the general behavior of the human body when submitted to different environments, and the analysis that follows is always valid (for the model adopted in this article).

## 4. Concluding Remarks

Several authors [10,11,12,13,14,15,16,17,18,19,20,21,22,23,24,25] performed the exergy analysis in the human body aiming at obtaining correlations of points of minimum $B_{d}$ with thermal comfort conditions. A distinguished feature of this analysis is the modification of the person anatomy (fat and muscle masses) and the direct comparison between the exergy destruction rate (and the exergy transfer rate to environment) with PMV and PPD. From the range analysed it is possible to conclude that:

- The effects of the body composition were successfully related to exergy parameters. It was noticed that the body metabolism influences the exergy destruction and the exergy transfer to environment, increasing both terms. This relation is evident since an increase in the muscle mass causes a higher exergy index increase than the same increase in the fat mass.
- The results obtained in this article demonstrate that the area normalization is a good generalization of the human body response when submitted to different environments. Therefore, the exergy analysis and its relation with PMV and PPD indexes may be used as a general tool to assess thermal comfort conditions.
- Points of minimum destroyed exergy do not always occur for the thermal comfort conditions. It was obtained that when the relative humidity is over 70% the destroyed exergy is minimal for PMV=0, but when the humidity is bellow 70% the destroyed exergy is close to its minimum value, but only the exergy transfer to environment is minimal for the thermal comfort conditions.
- For environments considered “cold” (PMV<0) it was shown that the relation between the exergy destruction rate and the predicted mean vote is independent of humidity.
- Again, for environments considered “cold” (temperatures lower than thermal neutrality), PMV is zero (or around zero) when $B_{d}$ is at its minimal.
- For the so called “hot” environments (temperatures higher than thermal neutrality) the thermal comfort condition is obtained when both $B_{d}$ and $B_{env}$ are minimal or around minimum points, demonstrating the results obtained by [24].

## Figures and Tables

**Table 1:** Layers and blood properties, based on [30,31,32,33,34].

Layer	ρ (kg/m${}^{3}$)	${\dot{M}}_{\mathrm{bas}}^{{}^{\prime \prime \prime }}$ (W/m${}^{3}$)	*k* (W/m·K)	$c_{p}$ (kJ/kg·K)	$w\dot{1}0^{6}$ (m${}_{b}^{3}$/m${}_{t}^{3}\cdot$s)
Core	1035	2629	0.5038	2.679	4157.5
Muscle	1006	684	0.5100	3.800	542.5
Fat	853	368	0.2100	2.300	76.7
Skin	1006	368	0.4700	3.680	361.7
Blood	1059	0	0.4700	3.850	-

## Abbreviations

The following abbreviations are used in this manuscript:

RQ	Respiratory coefficient
PMV	Predicted mean vote
PPD	Predicted percent dissatisfied

## Nomenclature

*A*	Area (m${}^{2}$)
*B*	Exergy rate (W)
*b*	Exergy per unity of mass (kJ/kg)
bm	Hole body mass (kg)
$c_{p}$	Specific heat (kJ/kgK)
*E*	Energy (W)
*H*	Enthalpy rate (W)
*h*	Specific enthalpy (kJ/kg)
*k*	Thermal conductivity (W/m·K)
*L*	Model height (m)
*m*	Mass (kg)
*M*	Metabolism (W)
*p*	Pressure (kPa)
*Q*	Heat transfer rate (W)
*q*	Heat transfer (W)
*R*	Gas constant (kJ/kgK)
*r*	Layer radius (m)
*s*	Specific entropy (kJ/kgK)
*T*	Temperature (°C)
*t*	Time (s)
*V*	Volume (m${}^{3}$)
*w*	Blood perfusion (m${}_{blood}^{3}$ / (m${}_{tisseus}^{3}$s))
*W*	Performed power (W)

## Greek letters

ρ	Density
ϕ	Relative humidity

## Subscripts

b	blood
bas	basal
c	core
carb	carbohydrate
conv	convection
d	destroyed
e	vaporization
env	environment
exp	expired
f	fat
lip	lipid
fg	vaporization
M	metabolism
m	muscle
prot	protein
rad	radiation
resp	respiration
s	skin
v	vapour
∞	conditions far from the body

## Superscripts

0	Thermal neutrality condition
$\dot{}$	Rate
${}^{\prime \prime \prime }$	Per unity of volume
